# The Study of the Transport Mechanism of Isorhynchophylline in Liver

**DOI:** 10.1155/2022/3867323

**Published:** 2022-01-19

**Authors:** Zhixian He, Jinyue Wang, Xing Wang, Yv Dong

**Affiliations:** School of Medicine, Southwest Jiaotong University, No. 111, Chengdu North 2nd Ring Road, Chengdu, Sichuan 610003, China

## Abstract

To investigate the transport mechanism of isorhynchophylline (IRN) by using the specific inhibitors of organic cation transporters (OCTs) and organic anion transporting polypeptides (OATPs) and attempt illustrate the metabolic mechanism of IRN in the liver. All animals were randomly divided into three groups: control group (only inject IRN), RIF group (inject IRN and rifampicin), and ADR group (inject IRN and adrenalone). The control group was injected with IRN via the caudal vein. The RIF group was injected with rifampicin (RIF) by gavage, and after 1 h, IRN was injected into the caudal vein. Similarly, the ADR group received adrenalone by the caudal vein, and after 0.5 h, IRN was injected into the caudal vein. Thereafter, blood samples were obtained by the heart punctures at 90 min, 180 min, and 300 min following drug administration. Rats were sacrificed at 300 min after drug administration; then, the liver tissue was harvested. The level of IRN was measured by using high-performance liquid chromatography (HPLC), and the Kp values were calculated. After RIF administration (OATPs inhibitors), the Kp value of IRN was slightly decreased when compared with that of the control group. Meanwhile, the Kp value of IRN was dramatically reduced compared to that of the control group following ADR administration (OCTs inhibitors). The results suggested that OCTs have mainly participated in the hepatic uptake process of IRN.

## 1. Introduction

**Figure 1:**
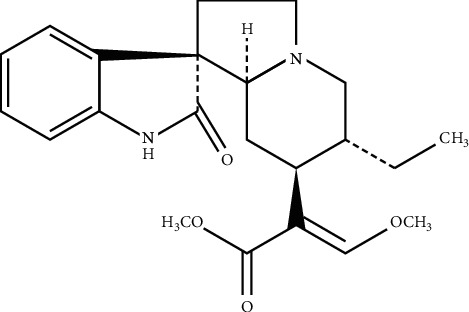
The chemical structure of IRN.

Traditional Chinese medicine (TCM), as a system of theories and therapeutic strategies, is usually used to prevent, diagnose, treat, and regulate the function of the human body [1]. TCM is mainly composed of botanical drugs, animal medicine, and mineral medicine, and its history can be traced back to over 2000 years [2]. According to the World Health Organization's report, TCM is used as primary health care in most countries, and approximately one-fourth of prescription medicines worldwide are derived from TCM [3]. Moreover, throughout the world and importantly in China, TCM is a leading complementary and alternative medicine manner and plays an increasingly indispensable role in the remedy of plentiful diseases in international medical practice [4]. In addition to being used directly as therapeutic agents for the prevention and treatment of disorders or diseases, medicinal plants are also used as influential resources for pharmacological research [5]. With the expanding prevalence and serviceability of medicinal products, the underlying molecular mechanism of TCMs has drawn more and more attention in recent years. Without any doubt, ascertaining the composition and biological activity of TCMs are essential for their safe and effective use. Isorhynchophylline (IRN, the chemical structure is shown in Figure 1) is a fundamental tetracyclic oxindole alkaloid component abundantly present in *Uncaria* species and also the central active chemical ingredient for its biological activities [6]. Consistent with the pharmacological activities, IRN could block the activity of calcium release from intracellular stores, exert protective roles against the ischemia, glutamate, or dopamine-induced damage or death [7], and lowered blood pressure against ischemia-induced neuronal damage [8]. Currently, IRN was extensively used to treat asthma, cancer, cirrhosis, diabetes, hypertension, stroke, and rheumatism [9]. Lee H found that IRN could extensively downregulate the expression level of antiapoptotic, proliferative, metastatic, and angiogenic genes and therefore bring about apoptosis by activating caspase-3/-8/-9 and inhibit the migratory and invasive potential of tumor cells [10]. Additionally, research had demonstrated that IRN could mitigate the neurotoxicity induced by A*β*25–35 by regulating the PI3K/Akt/GSK-3*β* signaling pathway [11] and ameliorates cognitive impairment via modulating amyloid pathology, tau hyperphosphorylation, and neuroinflammation [12], enhancing the antioxidant status and anti-inflammatory effect by means of nuclear factor kappa B (NF-*κ*B) signaling [13, 14]. Accordingly, further exploring the pharmacokinetics and metabolism of IRN is conducive to its clinical applications. The present study aimed to investigate the uptake mechanism of IRN in the liver and attempt to illuminate the underlying mechanisms.

## 2. Material and Methods

### 2.1. Animals and Grouping

A total of 18 male Sprague–Dawley (SD) rats (weighing 200 ± 20 g) were obtained from the Center of Experimental Animals, Sichuan Province People's Hospital. All protocols were conducted in accordance with the NIH Guidelines for the Care and Use of Laboratory Animals and were approved by the guidelines of the Institutional Medical Experimental Animal Care Committee of Southwest Jiaotong University. The animals were housed two-four per cage in temperature- and humidity-controlled cages and ventilation-controlled vivariums with a reverse 12 h light/dark cycle. Pellet chow and water were available ad libitum. The animals were randomly divided into three groups: control group (only inject IRN), RIF group (inject IRN and rifampicin), and ADR group (inject IRN and adrenalone).

### 2.2. Chemical Reagent Preparation

#### 2.2.1. Allocation of IRN

Isorhynchophylline (IRN, purity ≥ 98%) was purchased from the National Institute for the Control of Pharmaceutical and Biological Products (110807–200205, Beijing, China). To determine the appropriate dose of IRN, a preliminary experiment was performed by injecting the mice. When the dose is greater than 40 mg·kg^−1^, it can result in serious illness or death in mice. Accordingly, the usage dose of rats was 27.8 mg·kg^−1^ by calculating based on Table 1. Then, the volume of IRN was computed according to the formula: drop volume = drop speed (4 *μ*L·h^−1^·g^−1^) × time × weight.

#### 2.2.2. Allocation of Rifampicin (RIF) and Adrenalone (ADR)

The suitable dose of RIF (Shanghai Miriel Chemical Technology Co. Ltd, China) and ADR (Shanghai Miriel Chemical Technology Co. Ltd) is 30 mg·kg^−1^ and 20 mg·kg^−1^ by consulting literature materials [15], respectively. Then, the solution of RIF and ADR was separately configurated using saline and 30% PEG400.

### 2.3. Stock Solutions Preparation

#### 2.3.1. Reference Solutions

Reference solutions were prepared at a concentration of 2 mg·mL^−1^ using 10 mg IRN containing moderate methanol.

#### 2.3.2. IS Solution

10 mg carbamazepine (Chengdu Kelon Chemical Reagent Co. Ltd, China) was weighed and added moderate amounts of methanol to produce 1 mg·mL^−1^ standard solution. All solutions were stored at 4°C.

### 2.4. Drug Administration and Samples Harvest

All the animals were acclimated to pathogen-free laboratory conditions for 3 days. Then, rats were fasted for 12 hours before the experiment. The control group was injected with IRN (4 *μ*L·g^−1^·h^−1^) into the caudal vein. Meanwhile, the RIF group was given rifampicin by gavage for 1h, then IRN (4 *μ*L·g^−1^·h^−1^) was injected into the caudal vein. Similarly, the ADR group received adrenalone via the caudal vein for 0.5 h, then IRN (4 *μ*L·g^−1^·h^−1^) was injected into the caudal vein. Thereafter, blood samples were obtained by the heart punctures at 90 min, 180 min, and 300 min following drug administration. Rats were sacrificed at 300 min after drug administration, then the liver tissue was harvested. The blood samples and liver tissue were stored at −20°C.

### 2.5. Sample Preparation

#### 2.5.1. Blood Samples

0.1 mL blood samples were mixed with 10 *μ*L of standard solution. The solution was centrifuged (3,500 r·min^−1^) for 10 min after adding 0.4 mL methanol, and then, the supernatant was extracted. Afterwards, the supernatant was dried using nitrogen on a water bath at 60°C and dissolved with phosphate buffer saline (PBS) and then centrifuged (10,000 r·min^−1^) for 10 min to obtain the supernatant once again.

#### 2.5.2. Liver Tissue

Mix the liver tissue and PBS thoroughly. Then, the solution was centrifuged (3000 r·min^−1^) for 10 min, and the supernatant was extracted. Then, the tube was added with 10 *μ*L standard solution and 0.4 mL methanol. The solution was mixed by using a vortex mixer, standing for 2 h. Thereafter, the mixed solution was centrifuged (3,500 r·min^−1^) for 10 min. The supernatant was extracted and then dried using nitrogen on a water bath at 50°C and dissolved with PBS and then centrifuged (10,000 r·min^−1^) for 10 min to obtain the supernatant once more.

#### 2.5.3. Chromatographic Conditions and Method Validation

The analysis instrumentation consisted of an Agilent 1260 Infinity system (Agilent Technologies, USA) equipped with a G7114A UV-vis detector. Chromatographic separation was performed using a Waters™ C18 (4.6 × 250 mm, 5.0 *μ*m) analytical column at 30°C. The mobile phase consisted of methanol and water in the proportion of 70 : 30 (v/v). The pH was adjusted to 8.0 by using triethylamine, the flow rate was set to 0.8 mL/min, the column temperature was set at 30°C, and the sample injection volume was 20 *μ*L.

#### 2.5.4. Method Qualification and Sample Analysis Procedure

Analytical validation was in accordance with the recommendations of the International Conference on Harmonization guidelines [16]. The following characteristics were considered for validation: linearity, precision, accuracy, and robustness.

### 2.6. Selectivity and Specificity

The selectivity of the method was confirmed by injecting the blank plasma sample, reference solutions, and standard solution, respectively. Then, the specificity was evaluated by comparing the retention time of the analyte in different solutions. Monitoring the signals of m/z transition for qualifier ions could certify the presence of IRN.

### 2.7. Calibration Curve, Accuracy, and Precision

Quality control samples and standards were used for batch acceptance. The qualification run included duplicate calibration curves at six concentrations (2000, 1000, 400, 250, 200, and 50 *μ*g·mL^−1^) and quality control at three concentrations (2000, 500, and 100 *μ*g·mL^−1^). The acceptance criterion of the qualification run was within ±25% of precision and accuracy. The calibration curve (*y* = *ax* + *b*, where *x* = concentration, *y* = peak area ratio, *a* = slope, and *b* = intercept) was established via using the weighted regression method and defining the peak area ratios as functions of the theoretical concentrations. Additionally, two blank plasma samples were in the set.

The precision of the method was evaluated including intraday precision and interday precision. The relative standard deviation (%RSD) of the peak area from three concentrations prepared on the same day was used to calculate the intraday precision, and the %RSD of peak areas of three concentrations over three days was utilized to count the interday precision.

Accuracy was determined by the percentage recovery value. Three sets of samples were measured consisting of IRN standard solutions at concentrations of low (100 *μ*g·mL^−1^), medium (500 *μ*g·mL^−1^), and high (2000 *μ*g·mL^−1^) in 70% aqueous acetone. The assay was conducted over three consecutive days, and absolute recovery was counted following extraction and analysis of all sample sets.

### 2.8. Recovery

Extraction recovery of IRN was detected by comparing the peak areas of the extracted samples to the unextracted standard solutions. Briefly, 0.1 mL of acetonitrile was added to 0.1 mL of the blank plasma and the mixture was vortex-mixed for 1 min. Then, reference solutions (2000 *μ*g·mL^−1^) and standard solution (1000 *μ*g·mL^−1^) were added to obtain different concentrations including 2000, 500, and 100 *μ*g·mL^−1^. The supernatant was extracted and analyzed following centrifuging at 10,000 g for 5 min. The peak area (representing 100% recovery) was compared to that of the extracted black samples.

### 2.9. Stability of Samples

The system suitability was tested by three replicate analyses (including 2000, 500, and 100 *μ*g·mL^−1^). To measure the system performance, the suitability parameters were separately investigated in room temperature and freeze-thaw conditions. Briefly, the stability of solutions was evaluated every 6 hours (4 times in total) at room temperature, and the relative standard deviation (RSD) was calculated. Moreover, the samples were frozen for 21 hours at −20°C, and then left at room temperature for 3 hours. Therefore, the same process was repeated three times, and the RSD values were separately calculated.

### 2.10. Application to Pharmacokinetic Study

The validated assay method was applied to investigate the pharmacokinetic characteristics of IRN in rats receiving tail vein injection at a dose of 2.5 mL·kg^−1^. Rats were euthanized, and blood samples were collected by heart puncture at 90, 180, and 300 min after injection. Using the validated HPLC method as described above, the plasma samples were processed and analyzed. A concentration-time profile was constructed, and relevant pharmacokinetic parameters were calculated.

## 3. Results

### 3.1. Method Validation

#### 3.1.1. Selectivity and Specificity

**Figure 2:**
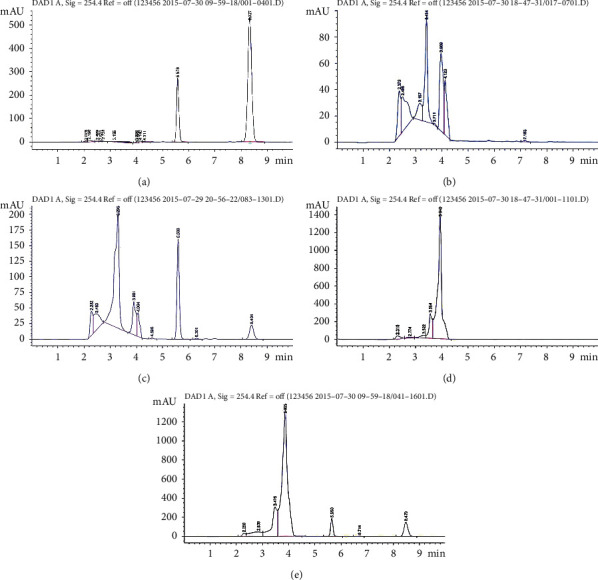
Method validation concerning selectivity and specificity. (a) The chromatograms of blank samples consisting of carbamazepine (5.578) and IRN (8.327), (b) the chromatograms of plasma samples in rats without any treatment, (c) the chromatograms of plasma samples in rats injected with carbamazepine and IRN, (d) the chromatograms of the liver tissue in rats without any treatment, and (e) the chromatograms of the liver tissue in rats injected with carbamazepine and IRN.

No significant peak was detected in the blank plasma processed with the above procedure. The results showed that this method had a better separation and specificity, and the endogenous compounds were no interference to the target peak (Figure 2(a)–2(e)).

#### 3.1.2. Linearity and Sensitivity

The linearity and sensitivity of the method were measured, and the calibration curve was constructed according to the different concentrations of IRN.

**Figure 3:**
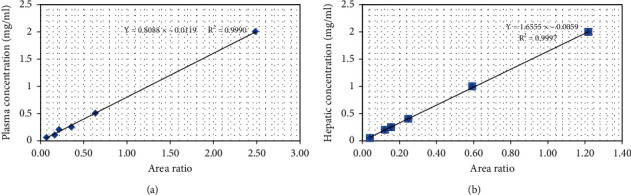
The standard curves of IRN. (a, b) Curves of the regression equation in the plasma and liver samples, respectively. *X* axis is the concentration, and *Y* axis is the ratio of the signal intensity of test compounds.

The standard curves are expressed as “*y* = *ax* + *b*” (*x* is the concentration and *y* is the ratio of the signal intensity of test compounds, and the parameters of *a* and *b* are the corresponding slope and intercept, respectively). The regression equation of the line in the plasma sample was obtained (*y* = 0.8088x − 0.0119), and the regression coefficient was greater than 0.99 (Figure 3(a)). Similarly, the regression equation of the line in the liver sample was acquired (*y* = 1.6555x − 0.0059), and the regression coefficient was greater than 0.99 (Figure 3(b)).

#### 3.1.3. Precision and Accuracy

The precision and accuracy of the assay method for IRN administration are summarized in Tables 2 and 3, respectively. To evaluate the method precision, standard solutions and reference solutions (2000, 500, and 100 *μ*g/mL) were prepared in triplicate and analyzed on the same day (repeatability) and in three consecutive days (intermediate precision), respectively.  Table 2 showed the precision. The maximum RSD value was 9.54%, which lower the upper limit values (10%). The accuracy was presented as percent deviation, and the values ranged from 90% to 102% (Table 3). Also, the RSD values of repeatability was 2.88% (plasma) and 1.68% (liver) (Table 4). These results suggested that this method was accurate, precise, and reproducible for the quantification of IRN in the rat plasma and liver.

#### 3.1.4. Recovery

The extraction method yielded a recovery of 95.5% and 105% for IRN at different drug administrations, which indicated that the recovery rates were consistent over the calibration ranges (Table 5).

#### 3.1.5. Stability

The results showed that the RSD values were lower than 10% in freeze-thaw cycles, room temperature, and low temperature, which indicated that the IRN solutions could be stored at −20°C for at least 8 days (Table 6).

### 3.2. Drug Content in Liver Tissue and Plasma

The liver tissue and plasma were obtained to determine the level of IRN at 90 min, 180 min, and 300 min, respectively. The results suggested that the Kp value (ADR group: OCT inhibitor) was lower than the Kp value (RIF group: OATP inhibitor), and the Kp value (RIF group: OATP inhibitor) was lower than the Kp value (control group) (Table 7).

## 4. Discussion

IRN is abundantly present in the ethanol extract of *Uncaria rhynchophylla* and is the main active chemical ingredient for its biological functions [6]. Accumulating evidence revealed that IRN is a promising herb for disease treatment including asthma, cancer, cirrhosis, diabetes, hypertension, stroke, and rheumatism [9]. Generally, hydrophilic drugs are slowly and passively absorbed and distributed into the blood; then, the drugs are uptaken by the liver or across the intestinal wall, which may take place via various pathways [17]. IRN, as an alkaloid, is transported relying on numerous factors consisting of the pH both within and outside the cell membrane and the pKa value of drugs, which greatly affect the lipid solubility and ionization of IRN. OCTs play crucial roles in the handling of cationic drugs and endogenously synthesized organic cations [18]. Moreover, some studies indicated that OCTs are critical for the uptake and transport of drugs [19, 20], and the membrane transport proteins are obviously expressed in certain target tissues like liver, muscle, and adipose tissue [18, 21]. In this study, rats received the drug by injecting IRN into the veins of the tails. Then, the IRN level in the liver tissue and plasma was acquired and analyzed by using the HPLC method. The results showed that the concentration of IRN in the liver was slightly lower than that of the control group after rats were given RIF (OATPs inhibitor) by gavage. However, when ADR (OCTs inhibitor) was injected into the caudal vein, the concentration of IRN in the liver was significantly lower than that of the control group. Therefore, we deduced that OCTs may partly participate in the transport of IRN in the liver. Yet, it is not known whether OCTs reduced uptake directly and whether they contribute to a malfunction in the therapeutic effects of IRN. Currently, the studies about transporters mainly comprise genetically knocked out animals and specific inhibitors. Additionally, the specific inhibitors method has characteristics of rapidness, simplicity, and low cost, which investigates the drug transport by comparing the effect of drug metabolism. Collectively, an in-depth study of the underlying mechanism between the transporter and IRN is needed, and it would provide an essential basis for the clinical development and application of IRN.

## Figures and Tables

**Table 1:** Equivalent dose ratio in different species.

	Mouse	Rat	Cavy	Rabbit	Cat	Monkey	Dog	Human
Mouse	1.0	7.0	12.25	27.8	29.7	64.1	124.2	387.9
Rat	0.14	1.0	1.74	3.9	4.2	9.2	17.8	56.0
Cavy	0.08	0.57	1.0	2.25	2.4	5.2	10.2	31.5
Rabbit	0.04	0.25	0.44	1.0	1.08	2.4	4.5	14.2
Cat	0.03	0.23	0.41	0.92	1.0	2.2	4.1	13.0
Monkey	0.016	0.11	0.19	0.42	0.45	1.0	1.9	6.1
Dog	0.008	0.06	0.10	0.22	0.23	0.52	1.0	3.1
Human	0.0026	0.018	0.031	0.07	0.078	0.06	0.32	1.0

**Table 2:** Precision for different concentrations of IRN.

Concentration (*μ*g·mL^−1^)	The RSD for the intraday (%)		The RSD for the interday (%)	
	Liver	Plasma	Liver	Plasma
2000	3.36	2.76	7.59	2.55
500	5.58	4.03	6.43	9.54
100	4.50	4.51	5.58	8.66
Mean	4.48	3.76	6.53	6.91

**Table 3:** Accuracy for different concentrations of IRN.

Concentration (*μ*g·mL^−1^)	Plasma		Liver	
	Measured value (*μ*g·mL^−1^)	Accuracy (%)	Measured value (*μ*g·mL^−1^)	Accuracy (%)
2000	1980	99	2040	102
500	490	98	460	92
100	90	90	90	90
Mean	—	96	—	95

**Table 4:** Repeatability for different concentrations of IRN.

Number	Area ratio			RSD (%)
	1	2	3	
Plasma	0.29	0.28	0.28	2.88
Liver	0.24	0.22	0.23	1.68

**Table 5:** Recovery for different concentrations of IRN.

Concentration (*μ*g·mL^−1^)	Plasma		Liver	
	Measured value (*μ*g·mL^−1^)	Recovery (%)	Measured value (*μ*g·mL^−1^)	Recovery (%)
2000	1910	95.5	2100	105
500	480	96	510	102
100	103	103	100	100
Mean	—	98.2	—	102

**Table 6:** Stability of IRN under different conditions.

Concentration (*μ*g·mL^−1^)	Room temperature RSD (%)		Low temperature RSD (%)		Freeze-thaw cycles RSD (%)	
	Live	Plasma	Live	Plasma	Live	Plasma
2000	4.59	8.14	6.02	5.54	4.68	8.34
500	5.13	2.88	8.02	6.78	5.64	7.12
100	9.25	4.87	4.40	2.90	6.79	6.75
Mean	6.32	5.29	6.14	5.07	5.70	7.40

**Table 7:** IRN content of different groups in the liver tissue and plasma.

Groups	Plasma(mg·mL^−1^)			Liver (mg·mL^−1^)	Kp value	Kp value (mean)
	90 min	180 min	300 min			
Control group 1	0.2438	0.0204	0.0884	0.0336	0.3800	0.3985 ± 0.0472
Control group 2	0.0655	0.1007	0.0418	0.0159	0.3802	
Control group 3	0.1327	0.2867	0.0794	0.0299	0.3768	
Control group 4	0.5363	0.4693	0.0800	0.0298	0.3727	
Control group 5	0.1321	0.0990	0.0304	0.0147	0.4828	
Control group 6	0.3526	0.0909	0.0786	—	—	

RIF group 1	0.0751	0.2311	0.1862	0.0275	0.1475	0.2859 ± 0.1218
RIF group 2	0.3236	0.0083	0.0679	—	—	
RIF group 3	0.0413	0.4281	0.0600	0.0200	0.3340	
RIF group 4	0.5044	0.1171	0.0800	0.0301	0.3764	
RIF group 5	0.6505	0.0898	0.0109	—	—	
RIF group 6	0.1362	0.0616	0.0065	0.0473	—	

ADR group 1	0.1111	0.0601	0.0734	0.0365	0.4977	0.1920 ± 0.2038
ADR group 2	0.0700	0.5202	0.0700	0.0064	0.0918	
ADR group 3	0.0900	0.2470	0.0043	—	—	
ADR group 4	0.0109	0.3578	0.0715	—	—	
ADR group 5	0.1910	0.0104	0.0166	0.0098	0.0900	
ADR group 6	0.0012	1.9255	0.1615	0.0143	0.0885	

## Data Availability

The datasets used or analyzed in the current study are available from the corresponding author upon request.

## Abbreviations

IRN: Isorhynchophylline
OCTs: Organic cation transporters
OATPs: Organic anion transporting polypeptides
RIF: Rifampicin
HPLC: High-performance liquid chromatography
TCM: Traditional Chinese medicine
NF-*κ*B: Nuclear factor kappa B
SD: Sprague–Dawley
PBS: Phosphate buffer saline
RSD: Relative standard deviation.
